# Physics-Based Predictive Modeling of Gravity-Induced Sagging in Support-Free Pellet Additive Manufacturing

**DOI:** 10.3390/polym17212858

**Published:** 2025-10-27

**Authors:** Alessio Pricci

**Affiliations:** 1 Department of Mechanics, Mathematics and Management (DMMM), Polytechnic University of Bari, Via E. Orabona 4, 70125 Bari, Italy; alessio.pricci@poliba.it; 2 Interdisciplinary Additive Manufacturing (IAM) Laboratory, Polytechnic University of Bari, Viale del Turismo 8, 74123 Taranto, Italy

**Keywords:** pellet additive manufacturing, computational fluid dynamics, support-free 3D printing, non-Newtonian flow

## Abstract

The fabrication of support-free structures in pellet additive manufacturing (PAM) is severely limited by gravity-induced sagging, a phenomenon lacking predictive, physics-based models. This study introduces and validates a numerical model for the thermofluid dynamics of sagging, aiming to correlate process parameters with filament deflection. A predictive finite element (FE) model incorporating temperature-dependent non-Newtonian material properties and heat transfer dynamics has been developed. This was validated via a systematic experimental study on a desktop-scale PAM 3D printer investigating nozzle temperature, printhead speed, screw speed and fan cooling, using polylactic acid (PLA) as a printing material. Findings show that process parameter optimization can reduce bridge deflection by 64.91%, with active fan cooling being the most dominant factor due to accelerated solidification. Increased printhead speed reduced sagging, whereas higher screw speeds and extrusion temperature showed the opposite effect. The FE model accurately replicated these results and further revealed that sagging ceases once the filament cools below its minimum flow temperature (approximately 150–160 °C for PLA). This validated model provides a robust foundation for tuning process parameters, unlocking effective support-free 3D printing in PAM.

## 1. Introduction

Additive manufacturing (AM) is a transformative technology with far-reaching implications across multiple industries, including aerospace, healthcare and automotive industries. The ability to fabricate complex geometries layer-by-layer allows AM for unprecedented design freedom, rapid prototyping and reduced material wastage [1,2].

Among the various AM techniques, material extrusion (MEX) stands out as one of the most widely adopted due to its simplicity and cost-effectiveness.

Different types of MEX have been proposed in recent years. Fused filament fabrication (FFF) is the most widespread MEX technology. In FFF, a filament of thermoplastic or elastomeric material is heated above its melting point (or, equivalently, at a temperature conveniently higher than the glass transition, for amorphous materials) into a heated cartridge. Then, the molten material is deposited on the build plate following a layer-by-layer pattern up to the completion of the final part.

FFF suffers from operational and scalability drawbacks, including risk of filament buckling at high feeding speeds and inherent limits on printing volume, which is generally below 1 m^3^.

These materials can be also 3D printed on middle to large scales by directly extruding the pelletized feedstock. The corresponding MEX technique is called pellet-based additive manufacturing (PAM) or fused granular fabrication. It is based on the employment of a vertically arranged single-screw extruder placed inside a heated barrel, to convey, melt and meter pellets, which are then extruded through a nozzle on a build plate layer-by-layer, similarly to FFF [2].

PAM offers a lower environmental impact by eliminating the energy-intensive intermediate step of producing filament from pellets, reducing the overall processing steps from two melting cycles (i.e., pellet-to-filament and then filament-to-part) to a single one [3]. Furthermore, large-scale PAM significantly enhances productivity and part size compared to FFF, allowing for printing volumes that can reach 7 m^3^ and increasing mass flow rates by up to 200× if compared to FFF [4,5].

**Figure 1:**
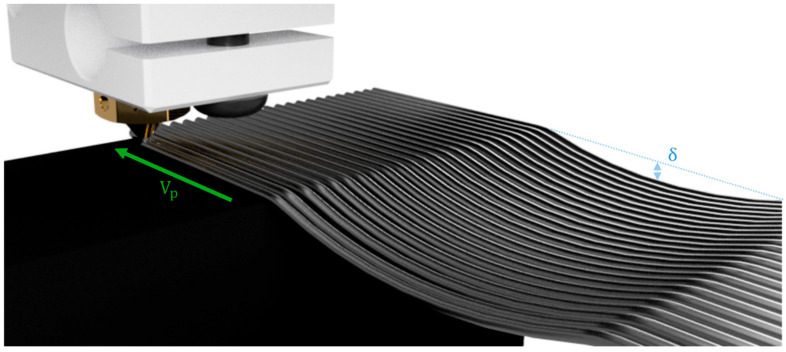
Graphical representation of support-free 3D printing. $V_{p}$: printhead speed; δ: maximum deflection of the overhanging structure.

However, one of the weak points of PAM is the impossibility of switching from one material to the other in most commercial systems. This limitation is particularly important when trying to 3D print structures characterized by overhangs, for which, generally, support structures should be used. Therefore, the possibility of 3D printing on large scales without the use of any support material is of interest (see Figure 1).

The amount of sagging due to gravity is also much more severe on large scales because of the higher amount of material being delivered in the unit of time. This aspect, combined with the increased nozzle diameters and low thermal conductivity of thermoplastic materials, leads to strands characterized by higher volume-to-area ratios compared to FFF.

Within the context of Industry 4.0, the minimization of material waste during manufacturing is also paramount [2]; this requirement further justifies the design of functional parts which can be 3D printed without the use of any support, since these are generally considered as manufacturing wastes. In small-scale 3D printing and especially in FFF, there are different possibilities when it comes to producing support-free structures.

Methods for achieving support-free 3D printing in MEX processes are often centered on optimizing the fabrication path and local printing conditions. One established technique is object partitioning, where complex models are decomposed into support-free sub-parts that are then printed sequentially [6,7]. This approach is significantly enhanced by multi-axis robotic systems, which utilize their high degrees of freedom to navigate complex geometries and build intricate structures in a collision-free manner [8,9,10,11]. However, these solutions are characterized by a high level of complexity in terms of both the algorithms and hardware to be used.

Similarly, manufacturing solutions to unlock 4D printing of complex structures made up of liquid crystal elastomers involve the use of extrusion-rolling systems as a further axis to enable the production of coil shapes [12].

Other solutions widely used for thermoset materials rely on the use of thixotropic baths to 3D print polymers [13,14,15]. These methods allow us to achieve superior part accuracy at the expense of the need for sacrificial support materials whose rheology must be carefully tuned.

Research into zero-support MEX 3D printing investigates the printable limits (e.g., the longest support-free printable length), and these investigations depend on the exact extrusion technique being employed; indeed, studies on conventional FFF focus on defining thresholds to minimize support waste [16,17], while investigations in large-scale PAM emphasize reducing material sagging through process control like additional cooling and precise calibration between extrusion flow rate and building speed [18].

In the framework of AM, numerical methods are gaining increasing attention because of their ability to capture the process–material relationship. Computational fluid dynamics (CFD) models have been extensively used to investigate the single- and multi-layer deposition of thermoplastic strands on the build plate.

The first investigations dealt with the influence of printing speed and layer height on the final strand shape in FFF, where accurately modeling the material’s non-Newtonian rheology was crucial to capturing the cross-sectional shape of 3D printed strands well [19,20,21]. However, the scale-up to nozzle sizes typical of PAM often reduces the significance of these non-Newtonian effects, allowing effective simulations of single-strand deposition using a simple Newtonian fluid assumption [22]. Ultimately, CFD is a powerful tool for process optimization, ranging from minimizing extrusion defects and optimizing printhead motion [23] to studying meso-structure formation, including porosity [24] and the effectiveness of post-processing techniques [25].

Although most of the literature related to the numerical simulation of MEX deals with the deposition of thermoplastic material on the build plate, little has been carried out with respect to support-free 3D printing, where the effective reduction in gravity-induced sagging remains an overwhelmingly empirical challenge, lacking predictive computational models.

To the best of the author’s knowledge there is no predictive physics-based mathematical model aimed at describing the influence of process parameters on the deflection observed in support-free 3D printing, especially when it comes to PAM.

This work presents the first numerical model for the thermofluid dynamics of bridging in PAM, considering non-Newtonian features, temperature-dependent thermodynamic properties and heat transfer with the surrounding environment.

The remainder of the research paper is organized as follows: First, the rheological and thermodynamic properties of the investigated PLA are introduced in Section 2, together with the experimental setup, design of experiment and numerical framework. Then, experimental and numerical results are compared and discussed in Section 3. Finally, Section 4 details the conclusions and outlines further work.

## 2. Materials and Methods

### 2.1. Material Characterization

NatureWorks Ingeo 3251D polylactic acid (PLA) has been used to investigate the effect of different printing parameters on the possibility of minimizing bridging in PAM.

The data required to conduct computational analyses included the following:

- Density: expressed as a function of temperature;
- Rheology: dynamic viscosity expressed as a function of temperature and shear rate;
- Thermodynamics: thermal conductivity (k) and specific heat capacity ($c_{p}$), whose values depend on temperature.

**Figure 2:**
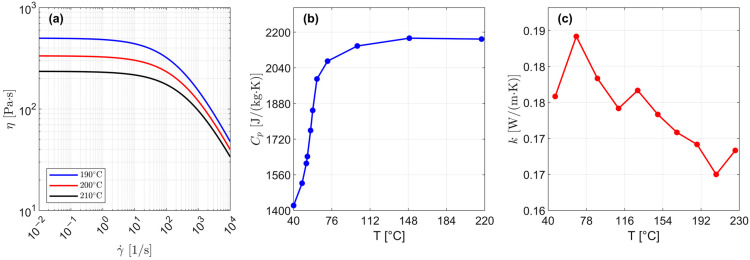
Material properties of the PLA 3251D: (**a**) rheological flow curves measured at various printing temperatures, (**b**) heat capacity as a function of temperature, and (**c**) thermal conductivity as a function of temperature.

The material properties implemented in the FE simulations are represented in Figure 2.

Material properties have been extracted through the software Autodesk Moldflow v.21. In particular, the specific heat ($c_{p}$) and glass transition temperature ($T_{g}$) were determined using differential scanning calorimetry (DSC). The measurements adhered to the ASTM E 1269 and ASTM D 3418 standards [26,27] for the measurement of $c_{p}$ and $T_{g}$, respectively. Samples, in pellet form, were subjected to a controlled cooling rate of 20 °C/min from 230 °C to 50 °C prior to the analysis.

Thermal conductivity (k) was measured using the transient line-source technique, following the specifications of ASTM D 5930 [28]. Prior to testing, the pellet-form samples were dried in a hopper dryer for 4 h at 70 C to achieve a low initial moisture content of 0.017%.

Rheological data expresses the non-linear relationship between dynamic viscosity (η) and both shear rate ($\overset{˙}{\gamma }$) and temperature (T). At very low shear rates (i.e., for $\dot{\gamma }<$ 11/s, as shown in Figure 2a), PLA behaves as a Newtonian fluid, while a power–law relationship (i.e., a straight line in the double logarithmic representation of $\eta =\eta (\dot{\gamma })$, see Figure 2a) can be observed at higher ones. The effective shear rate in a PAM process depends on (i) screw peripheral speed (N), (ii) nozzle diameter (D) and (iii) printhead speed ($V_{p}$).

A model to predict the material behavior in the metering zone of PAM barrel-screw systems depending on N has been proposed in [29]. However, the influence of process parameters when printing support-free structures has not been modeled yet; it therefore constitutes the main goal of this work.

### 2.2. Experimental Setup and Design of Experiments

A desktop-scale Direct 3D pellet extruder (Direct 3D s.r.l.) has been used to experimentally study the influence of the abovementioned printing parameters on the gravity-induced sagging of overhanging strands.

The PAM system consists of a 12.9 mm constant-pitch single-screw extruder placed inside a heated barrel. The pelletized thermoplastic is gradually heated above the melting point inside the screw vanes. Then, it is extruded through a 1.2 mm brass nozzle.

Custom-made G-Codes were designed to precisely control printing parameters when printing the overhanging structures. Four replications were made for each experimental condition.

Finally, overhangs have been post-processed in a MATLAB subroutine (MATLAB R2024a; MathWorks Inc., Natick, MA, USA) via the image-processing toolbox to find the maximum deflection ($\delta_{exp}$, see Figure 1).

The main process parameters which affect the amount of bridge deflection were nozzle temperature ($T_{N}$), printhead speed ($V_{p}$), screw peripheral speed (N) and fan state, namely its full activation speed or its shutdown (On or Off states, in the following).

Two levels (i.e., low and high) of each factor and a full factorial experiment design were chosen to study the influence of each process parameter and their mutual interaction on the amount of sagging (see Table 1).

### 2.3. Computational Model

The computational domain consisted of a double-clamped beam made up of viscous material. To fully capture the deformation of the viscous beam under the influence of its own weight, the software COMSOL Multiphysics v. 5.3 (COMSOL, Inc., Burlington, MA, USA) was employed.

The fluid is assumed to be purely viscous, i.e., the elongational effects are neglected. In addition, flow is assumed to be laminar since the Reynolds number remains below the unit for all process parameter combinations being investigated.

Consequently, laminar flow and heat transfer in fluid COMSOL modules were combined to define a comprehensive multi-physical model.

The governing equations solved to fully capture the transient gravity-induced deformation of the aforementioned viscous beam are as follows:

(1) $$\left\{\begin{array}{c} \frac{\partial \rho }{\partial t}+\nabla \cdot (\rho \vec{u})=0\\ \rho \frac{\partial \vec{u}}{\partial t}=-\vec{\nabla p}+\nabla \cdot \overset{̿}{\tau }+\rho \vec{g}\\ \rho \;c_{p}\frac{DT}{Dt}=-\vec{q}-\overset{̿}{\tau }:\overset{̿}{\nabla u}-p\left(\nabla \cdot \vec{u}\right) \end{array}\right.$$

These are the continuity, momentum and energy equations, respectively. In particular, t is the computational time, $\vec{u}$ is the fluid velocity vector, ρ is the fluid density, p is the fluid pressure, $\overset{̿}{\tau }$ is the viscous stress tensor, $\vec{g}$ is the gravity acceleration, $c_{p}$ is the specific heat, T is the extrusion temperature, and $\vec{q}$ is the heat flux.

**Figure 3:**
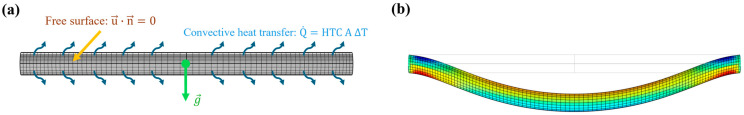
(**a**) Boundary conditions employed in the CFD model; (**b**) result of the application of the moving mesh method to track the deformation of the simulated support-free 3D printed bridge.

A schematic representation of the boundary conditions employed to solve the former system and the final deformed shape are represented in Figure 3.

In the momentum equation, the shear stress tensor $\overset{̿}{\tau }$ can be calculated as follows:

(2) $$\overset{̿}{\tau }=2\eta \left(\dot{\gamma }\right)\overset{̿}{D}$$

In the former expression, η, $\dot{\gamma }$ and $\overset{̿}{D}$ are the dynamic viscosity, shear rate and rate of deformation tensor, respectively. Shear rate is related to the effective amount of fluid deformation as follows:

(3) $$\dot{\gamma }=\sqrt{2\overset{̿}{D}:\overset{̿}{D}}$$

Because of the highly non-linear dependence of material rheology on shear rate and temperature (see Figure 2a), Cross-WLF rheological modeling was employed:

(4) $$\eta \left(\dot{\gamma }\right)=\frac{\eta_{0}}{{\left(1+\frac{\eta_{0}}{\tau^{*}}\right)}^{1-n}}$$

Here, $\eta_{0}$ is the zero-shear viscosity, whose dependence on temperature can be expressed with an Arrhenius-type relationship:

(5) $$\eta_{0}=D_{1}e^{-\frac{A_{1}\left(T-T^{*}\right)}{A2+T-T^{*}}}$$

$D_{1}$ is the zero-shear viscosity at a reference temperature $T^{*}$ and zero pressure. $A_{1}$ governs the slope of the viscosity–temperature curve and the overall temperature sensitivity. $\tau^{*}$ is the critical stress level at the transition to shear-thinning. n is the power–law index, defining the degree of shear-thinning in the high-shear rate regime (i.e., the lower the power–law index, the stronger the shear-thinning behavior). The reference temperature $T^{*}$ can be expressed as follows:

(6) $$T^{*}=T_{g}+D_{3}p$$

Here, $T_{g}$ is the glass transition temperature, $D_{3}$ a data-fitted coefficient and p the pressure.

The Data Fitting module available for Autodesk Moldflow was used to calculate the best fit for the remaining Cross-WLF parameters (i.e., $A_{1}$, $A_{2}$, $D_{1}$, $T^{*}$, n and $\tau^{*}$), which are reported in Table 2.

The expression for $\eta \left(\dot{\gamma }\right)$ given in Equation (4) was used, together with the shear stress tensorial expression of Equation (2), to model the viscous contribution of the molten thermoplastic and solve Equation (1).

To solve the energy equation, density ρ, specific heat $c_{p}$ and thermal conductivity k data were linearly interpolated between contiguous temperature intervals.

However, a complete model for sagging also requires the solution of the heat transfer between molten thermoplastics and the surrounding air.

Since the overhanging strand can be approximated as a cylinder whose diameter depends on process parameters (e.g., extrusion temperature T and printhead speed $V_{p}$) and geometry (nozzle diameter D), correlations from the literature can be used to model the heat exchanged by convection. These have proved to be efficient with respect to experimental data in the recent literature [25].

The heat transfer rate boundary condition has been applied to the air-to-thermoplastic interface, pointing outwards orthogonally to the boundary itself (see Figure 3a).

(7) $$\dot{Q}=HTC\;A\left(T-T_{0}\right)$$

Here, HTC is the heat transfer coefficient, A the air-to-thermoplastic interfacial area, T the local thermoplastic temperature and $T_{0}$ the surrounding air temperature (set to 25 °C in all computations). In this way, the heat transfer is solved locally for each computational cell belonging to the viscous beam geometry and for the exact PAM process parameters.

HTC was evaluated differently when considering 3D printing of overhanging structures with a fan in the On or Off state. In fact, in the former case, HTC is higher because of the influence of forced convection, while in the latter, heat is exchanged only by natural convection.

When considering forced convection, the following correlation can be applied to calculate the Nusselt number:

(8) $$Nu=0.3+\frac{0.62Re^{1/2}{Pr}^{1/3}}{{\left[1+{\left(0.4/Pr\right)}^{2/3}\right]}^{1/4}}{\left[1+{\left(\frac{Re}{282000}\right)}^{5/8}\right]}^{4/5}$$

Here, Re and Pr represent the Reynolds and Prandtl numbers, respectively.

When the fan is not activated (i.e., Off state), thermoplastics exchange heat with the surrounding air by natural convection. In this case, a different correlation applies [30]:

(9) $$\frac{2}{Nu}=\ln\left\{1+\frac{2}{{\left\{{\left[0.518Ra^{\frac{1}{4}}{\left[1+{\left(0.559/Pr\right)}^{\frac{3}{5}}\right]}^{-\frac{5}{2}}\right]}^{15}+{\left(0.1Ra^{1/3}\right)}^{15}\right\}}^{1/15}}\right\}$$

In addition to the aforementioned dimensionless parameters, Ra stands for the Rayleigh number.

In the former correlations, all fluid properties were evaluated at the mean film temperature:

(10) $$T_{film}=\frac{T_{N}+T_{0}}{2}$$

In this expression, $T_{N}$ and $T_{0}$ are the nozzle and surrounding air temperatures, respectively.

HTC has been evaluated through parameter-dependent values of thermal conductivity k, Nusselt number Nu and effective strand diameter $D_{eq}$, which was considered as the characteristic length:

(11) $$HTC=\frac{Nu\;k}{D_{eq}}$$

The calculation of $D_{eq}$ follows the mass conservation principle; this aspect will be shown in Section 3.4.

Finally, a free surface boundary condition has been applied to the air-to-thermoplastic interface:

(12) $$\vec{u}\cdot \vec{n}=0$$

A moving mesh approach was exploited to track the deformation of the computational grid, as gravity exerts its influence on the unsupported viscous thermoplastic strand.

The initial condition needed to solve the energy equation in Equation (1) was imposed in a way that initial temperature was set equal to the extrusion temperature (i.e., $T\left(t=0s\right)=T_{N}$), while zero velocity and pressure were initially set for fluid flow variables. The coupling of energy with the momentum equation is given by the viscous dissipation term (i.e., $\overset{̿}{\tau }:\overset{̿}{\nabla u}$ in energy equation, see Equation (1)).

First-order shape functions were used to solve velocity and pressure fields, and second-order ones for temperature. Simulations were performed on an Intel i7-13620H octa-core processor with 16 GB DDR5 5200 MHz RAM.

## 3. Results and Discussion

### 3.1. Three-Dimensional Printing and Analysis of Support-Free Structures

The Direct3D PAM 3D printer was used to produce overhanging strands on a base structure, where anchors were 20 mm distant from each other, following the design of experiment in Table 1. After 3D printing the overhanging strands under the process parameters highlighted in Table 1, the gravity-induced sagging was measured by post-processing images in a MATLAB subroutine via the Image Toolbox.

The 3D printed overhanging strands were weighed in each experimental condition with a Kern Emb 200-3 balance (resolution: 0.001 g; full scale: 200 g). The measured values, combined with an accurate measurement of the overall printing time, yielded the mass flow rates to be used as input data for the FE model, as will be outlined in Section 3.4.

Table 3 presents the mean deflection and mass flow rate values collected at varying process parameters.

### 3.2. Experimental Results—Main Effect Analysis

**Figure 4:**
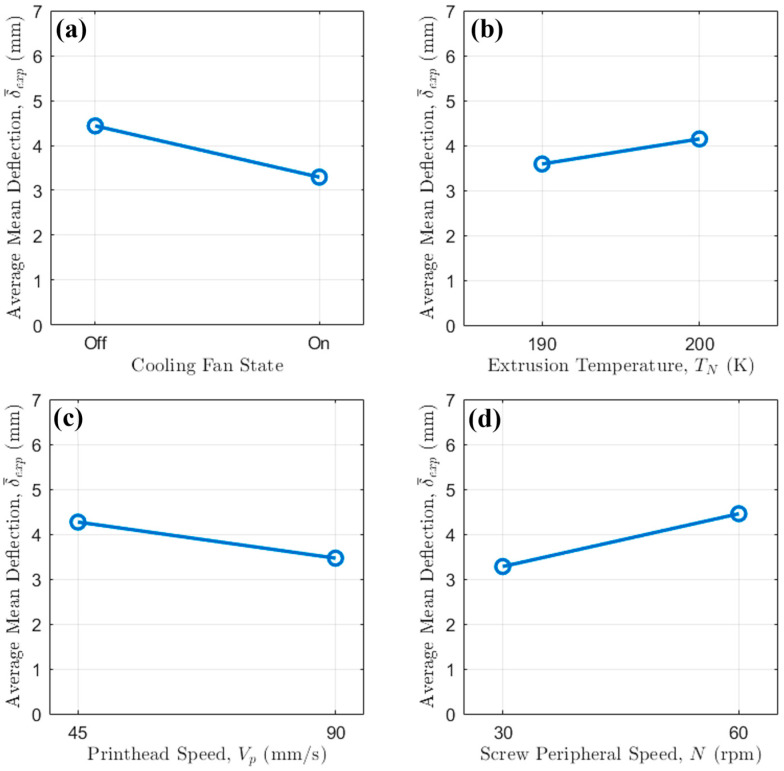
Main effect plot of (**a**) cooling fan state, (**b**) extrusion temperature, (**c**) printhead speed and (**d**) screw peripheral speed on strand average mean deflection.

For the abovementioned results, an analysis of the main effects was first conducted (Figure 4).

The results clearly indicate that active cooling via fan activation is the most dominant factor in minimizing bridge deflection, with screw peripheral speed also playing a significant role.

Across all tested conditions, the activation of the cooling fan resulted in a dramatic reduction in the average mean deflection. This observation underscores the critical importance of rapid thermal management in bridging 3D printing, especially at intermediate to large scales. Without active cooling and the additional heat transfer given by forced convection, the extruded polymer filament remains in a molten state for more time. Because of the lower dynamic viscosity, it is therefore highly susceptible to gravitational sag. The introduction of forced air convection via fan activation accelerates the solidification process, rapidly increasing the filament’s viscosity and freezing it in place shortly after deposition. This rapid quenching enhances the material’s ability to support its own weight over the bridge span, thereby drastically reducing deflection.

The influence of extrusion temperature (Figure 4b) is more nuanced and is strongly modulated by the fan’s activation. An increase in temperature from 190 °C to 200 °C leads to a slight increase in average deflection. This is consistent with polymer rheology, as a higher melt temperature corresponds to a lower viscosity, thus making the material more susceptible to sag.

The main effect plot for printhead speed (Figure 4c) reveals an inverse correlation with bridge deflection: increasing the speed from 45 mm/s to 90 mm/s consistently lowers the deflection. This behavior can be attributed to the reduced time the filament is suspended over the unsupported gap while the pellet extruded is still extruding at a mass flow rate in free air. At a higher $V_{p}$, the bridge is completed more quickly, affording the molten filament less time to deform under gravity before the structure is complete.

The screw peripheral speed N shows a strong relationship with bridge deflection (Figure 4d). Increasing N from 30 rpm to 60 rpm results in more severe deflections. This is due to the increase in mass flow rate with screw rotational regime; in fact, at higher N the mass flow rate delivered by the single-screw extruder is higher, resulting in a heavier filament strand. This increased weight amplifies the effect of gravity, leading to more pronounced sagging, especially when relying solely on natural convection (i.e., when the fan is Off).

Therefore, the most effective strategy to reduce sagging in bridging 3D printing involves (i) implementing additional cooling via fans, thus allowing for a quicker filament solidification, (ii) increasing printing speed and (iii) reducing screw peripheral speed to avoid extruding excessive quantities of molten thermoplastic in the free air.

### 3.3. Experimental Results—Interaction Analysis

**Figure 5:**
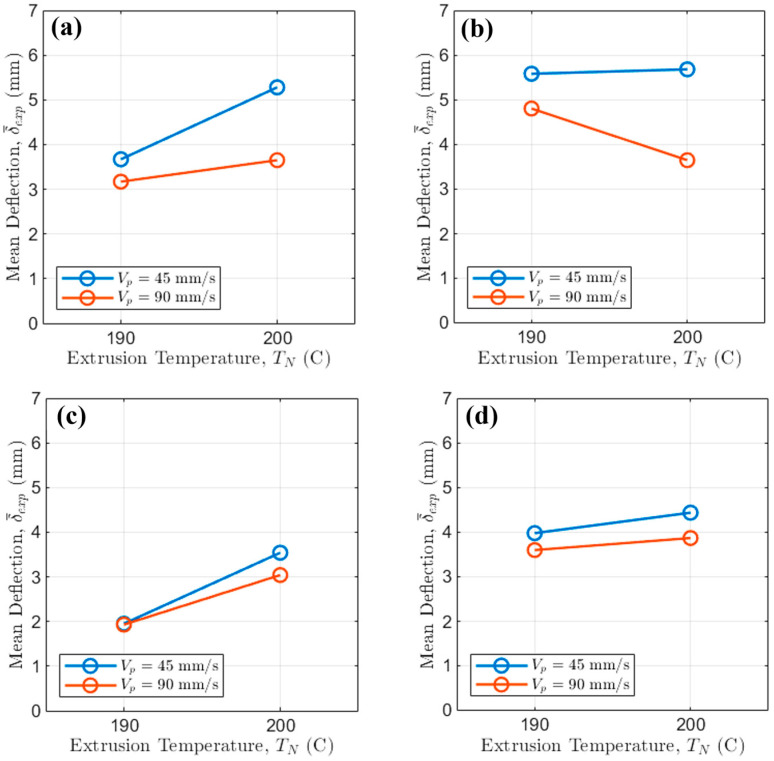
Interaction of printing speed and extrusion temperature when (**a**) the fan is Off and N = 30 rpm; (**b**) the fan is Off and N = 60 rpm; (**c**) the fan is On and N = 30 rpm; (**d**) the fan is On and N = 60 rpm.

The effect of $T_{N}$ on bridge deflection ${\bar{\delta }}_{exp}$ at different $V_{p}$ levels was first examined (Figure 5).

High $V_{p}$ (i.e., 90 mm/s) is highly effective, consistently resulting in lower deflection across all tested $T_{N}$, N, and fan settings. The overall maximum deflection (i.e., Figure 5b at $V_{p}$ = 45 mm/s) approaches ${\bar{\delta }}_{exp}$ ≈ 5.7 mm, while the minimum deflection (i.e., Figure 5c at $V_{p}$ = 90 mm/s) approaches ${\bar{\delta }}_{exp}$ ≈ 2 mm, demonstrating a potential reduction of over 64.91% driven by forced cooling and higher $V_{p}$.

The influence of $T_{N}$ is subtle: for the worst-case scenario (i.e., fan Off, N = 60 rpm), increasing $T_{N}$ from 190 °C to 200 °C increases ${\bar{\delta }}_{exp}$ by only about 0.4 mm, independently from $V_{p}$. Conversely, under the best conditions (fan on, N = 30 rpm), $T_{N}$ = 200 °C yields slightly lower deflections than $T_{N}$ = 190 °C for high $V_{p}$, underlining that rapid cooling dictates the outcome, thus overriding the minor viscosity changes caused by temperature within this tested range.

**Figure 6:**
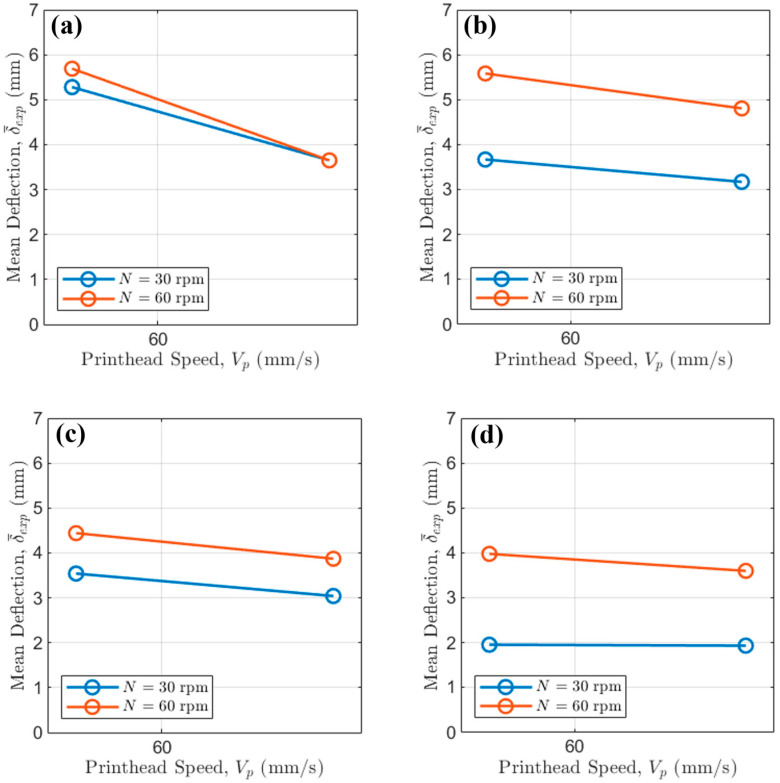
Interaction of printing speed and screw peripheral speed when (**a**) the fan is Off and $T_{N}$ = 200 °C; (**b**) the fan is Off and $T_{N}$ = 190 °C; (**c**) the fan is On and $T_{N}$ = 200 °C; (**d**) the fan is On and $T_{N}$ = 190 °C.

Then, the effect of $V_{P}$ on bridge deflection at different N levels was examined (Figure 6).

High values of $V_{P}$ (i.e., 90 mm/s) always reduce bridge deflection. For instance, at the high values of $T_{N}$, when the fan is Off (Figure 6a), switching from $V_{P}$ = 45 mm/s to $V_{P}$ = 90 mm/s reduces ${\bar{\delta }}_{exp}$ by approximately 2 mm regardless of N.

**Figure 7:**
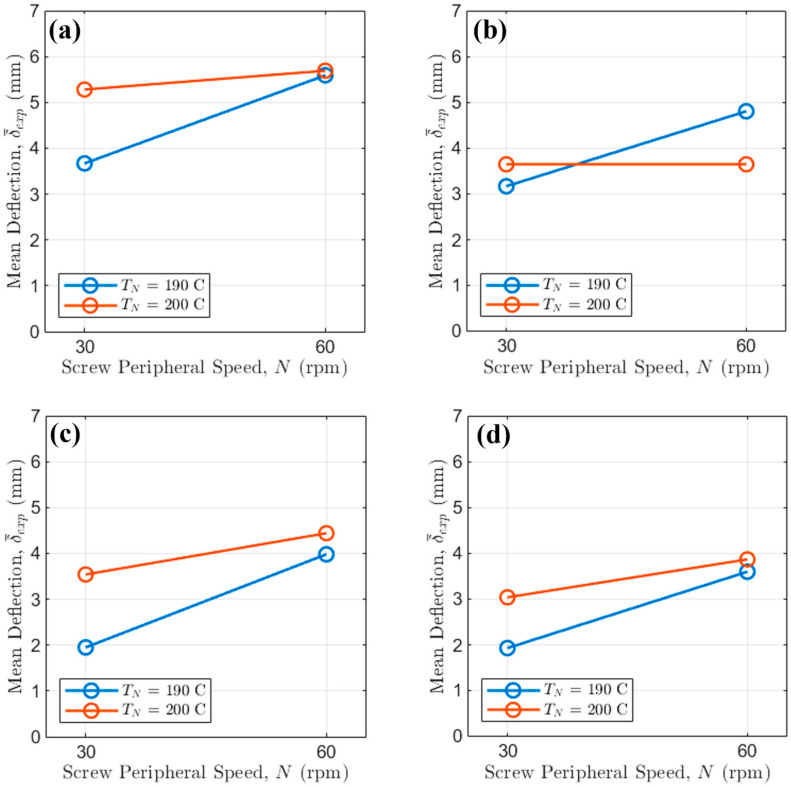
Interaction of extrusion temperature speed and screw peripheral speed when (**a**) the fan is Off and $V_{p}$ = 45 mm/s; (**b**) the fan is Off and $V_{p}$ = 90 mm/s; (**c**) the fan is On and $V_{p}$ = 45 mm/s; (**d**) the fan is On and $V_{p}$ = 90 mm/s.

Finally, the effect of N on ${\bar{\delta }}_{exp}$ for different values of $T_{N}$ is considered (Figure 7).

High screw rotational regimes (i.e., N = 60 rpm) consistently lead to higher deflection than lower ones because of the increase in mass flow rate. This is independent from the exact extrusion temperature, but the increase in ${\bar{\delta }}_{exp}$ is consistently higher at lower $T_{N}$.

As expected, the fan On curves are heavily compressed toward the bottom of the plot, generally showing ${\bar{\delta }}_{exp}$ values less than 4.5 mm. The clearest interaction is seen when the fan is Off. For $V_{P}$= 45 mm/s (slow printing), the difference between high and low N values is pronounced, with high N resulting in up to 0.7 mm higher deflection. This difference shrinks at high $V_{p}$, demonstrating that faster printhead speeds mitigate the negative effect of increased mass flow rate, related mainly to N. Critically, even with the fan active, maintaining a low N is essential: for $V_{p}=$90 mm/s (Figure 7d), the average deflection with N = 30 rpm is ${\bar{\delta }}_{exp}$ ≈ 3.5 mm, while employing N = 60 rpm results in ${\bar{\delta }}_{exp}$ ≈ 3.9 mm, highlighting that N still accounts for an important 0.4 mm difference in the minimal deflection achievable.

### 3.4. Computational Results

The cross-sectional dimensions of the overhanging strand depend on the experimentally measured mass flow rate, which varies with (i) screw peripheral speed N and (ii) extrusion temperature $T_{N}$. This mass flow rate was determined experimentally prior to conducting computational studies, as outlined in Section 3.1.

Once the mean mass flow rate $\bar{\dot{m}}$ has been determined for a given set of process parameters (see Table 3), the HTC value is calculated accordingly to Equation (8) or (9) in the case of forced or natural convection, respectively.

Then, the effective parameter-dependent cross-sectional diameter of the overhanging strand is calculated by estimating the mean diameter of the cylindrical extruded strand according to the mass flow rate definition (Equation (13)).

(13) $$D_{eq}=\sqrt{\frac{4}{\pi }\frac{\bar{\dot{m}}}{V_{p}}}$$

The approach based on considering a deforming circular cross-sectional shape is a good assumption when it comes to low gravity-induced sagging. For severe deflections, alternative methods based on fully three-dimensional CFD simulations should be used. However, the goal of this work is to find suitable combinations of printing parameters which reduce sagging. For that reason, the former assumption is consistently used in present applications of the FE model.

Prior to performing FE simulations under the same process parameters outlined in the design of experiment in Table 1, a mesh-independence study was conducted with respect to the following combination of process parameters: $T_{N}$= 190 °C, $V_{p}$= 45 mm/s, N=30 rpm and fan in the Off state. First, a structured O-grid mesh was used to discretize the left cross-sectional circular surface of the overhanging strand in its original state (i.e., not deformed by gravity). Subsequently, the mesh was swept from the left to the right end of the viscous beam. The number of intermediate elements in this last sweep meshing phase was varied to find the optimal value, i.e., the one which gives mesh-converged results with the shortest computational time (see Table 4).

The maximum deflection is always located at the center of the viscous beam, since gravity acts as a distributed load along its entire length. FE results show that using 20 elements provide mesh-converged results, since the relative error with respect to the finest mesh is below 1%, also achieving a computational time reduction of 58.33% with respect to the finest mesh.

**Figure 8:**
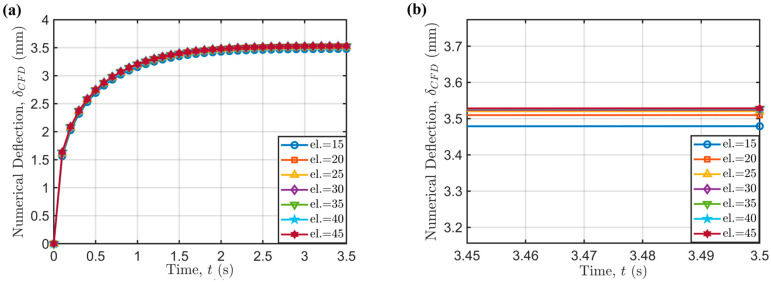
(**a**) Time-dependence of maximum bridge deflection ($\delta_{CFD}$) for different mesh resolutions (el.); (**b**) detail on the time-converged values.

The results at varying mesh resolutions were confirmed also with respect to the evolution of maximum deflection with time (Figure 8).

A direct comparison of experimental and numerical results in terms of strand deflection is given for the complete experiment design in Table 5.

As can be seen in Table 5, the proposed FE model describes the experimental results well under different values of the process parameters. This is especially true under the combination of process parameters which leads to less severe sagging. The opposite is also true, and it is due to some intrinsic model limitations, including the assumption of considering the cross-sectional shape as constant during filament sagging and neglecting the shrinkage.

In addition, the model is dynamic in terms of considering the evolution of sagging with time; however, a more accurate model should consider the viscous beam as one whose length is continuously defined over time through an incremental advancement of the printhead. In addition, higher-order shape functions can potentially improve model accuracy.

**Figure 9:**
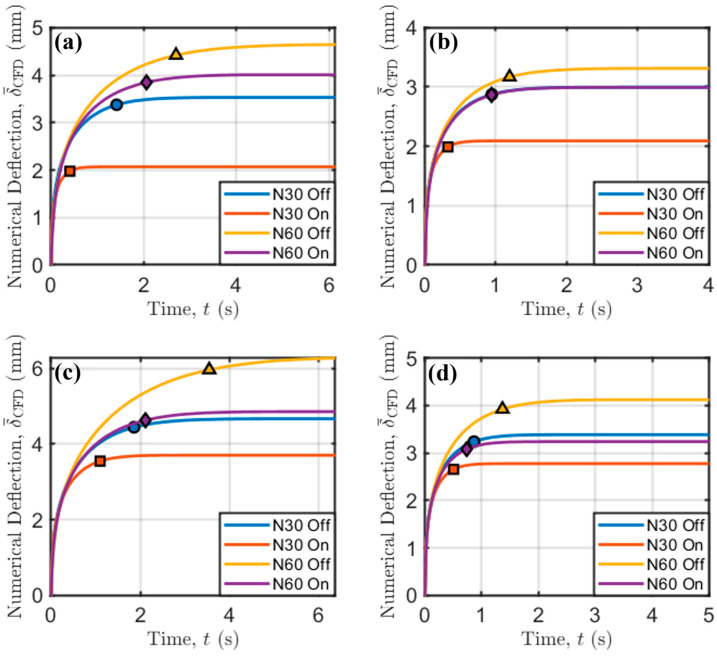
Temporal evolution of bridge deflection. (**a**) $T_{N}$ = 190 °C and $V_{p}$ = 45 mm/s; (**b**) $T_{N}$ = 190 °C and $V_{p}$ = 90 mm/s; (**c**) $T_{N}$ = 200 °C and $V_{p}$ = 45 mm/s; (**d**) $T_{N}$ = 200 °C and $V_{p}$ = 90 mm/s. Markers indicate the time needed to reach 95% of the final deflection.

To achieve a deeper understanding of how process parameters affect bridge deflection, one of the main strengths of numerical models is leveraged: their ability to provide insight into variables that are extremely difficult to monitor. In particular, the evolution of bridge sagging with time has been studied, as shown in Figure 9.

In addition, the times needed to reach 95% of final sagging for each combination of process parameters, as marked previously in Figure 9, are collected in the following Table 6.

At low printing speed and extrusion temperature (Figure 9a), the effect of increasing the screw speed (i.e., the flow rate and strand diameter according to Equation (13)) is an increase in the time needed to achieve time-independent sagging. However, the forced convection coming from fan activation can mitigate the time needed to reach a stable value because of the higher amount of heat exchange through the air-to-thermoplastic interface, motivated by the higher HTC when forced convection is exploited.

This trend is common to all other printing conditions (Figure 9b–d). Interestingly, at low extrusion temperature and high printing speed (Figure 9b), there is a compensation for the effects of increasing screw speed and activation of the fan, which have an opposite effect on bridge deflection. The curves relative to the evolution with time of these two cases overlap, and, at high extrusion temperature (Figure 9c,d), the curves remain very close. This indicates that the increase in mass flow rate delivered by the extrusion system, which also produces an increase in $D_{eq}$ (see Equation (13)), is compensated for by the fan activation. This is due to an increase in both HTC and the air-to-thermoplastic interface, both of which increase the amount of heat exchanged with the surrounding environment (see Equation (7)).

**Figure 10:**
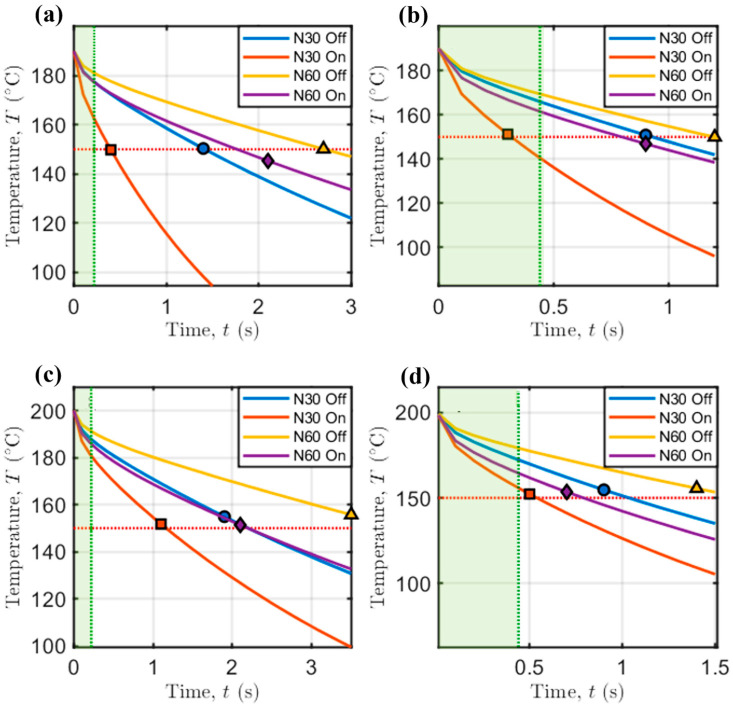
Temperature in the center of viscous beam as a function of time. (**a**) $T_{N}$ = 190 °C and $V_{p}$ = 45 mm/s; (**b**) $T_{N}$ = 190 °C and $V_{p}$ = 90 mm/s; (**c**) $T_{N}$ = 200 °C and $V_{p}$ = 45 mm/s; (**d**) $T_{N}$ = 200 °C and $V_{p}$ = 90 mm/s. Markers indicate the time needed to reach 95% of the final deflection, as depicted in Figure 9. Orange dotted line: T= 150 °C. Green dotted line: theoretical time needed by the printhead to complete the bridge.

The aforementioned considerations are confirmed by the slope of the curves depicting the decrease in temperature with time at the point of maximum sagging (Figure 10).

Interestingly, the time needed to reach 95% of the final sagging (see Table 6) is almost the one needed by PLA to reach a final temperature between 150 °C and 160 °C, which is the minimum material flowing temperature for this amorphous material. Below this temperature, the dynamic viscosity of the material becomes so high that it stops flowing, thus sagging less under the influence of gravity.

The vertical green lines in Figure 10 indicate the duration of bridge formation: since the support-free distance between the anchors is 20 mm, the theoretical time needed for the printhead to print the overhanging strands is 0.22 s or 0.44 s, when $V_{p}$ is equal to 45 mm/s or 90 mm/s, respectively (green intervals in Figure 10).

The case with $T_{N}$ = 190 °C, N = 30 rpm, $V_{p}$ = 90 mm/s and fan On (see Figure 10b) experiences a steeper temperature decrease to the aforementioned interval for PLA (i.e., 150–160 °C), which is also compatible with the time needed for bridge formation, falling in the green zone highlighted in Figure 10. This motivates the deflection being only 1.93 mm, which is the overall minimum gravity-induced sagging according to our experiments.

This design criterion is therefore recognized as potentially impactful when modeling support-free 3D printing: it is possible to print support-free structures characterized by less severe gravity-induced deflections when the time needed to print support-free structures is lower than that needed to cool the material below its melting point.

## 4. Conclusions

This work presents the first predictive, physics-based numerical model for the description of thermofluid dynamics of bridging in support-free PAM, focusing on correlating gravity-induced sagging with process parameters and material cooling rate. This model uniquely considers temperature-dependent material properties such as heat capacity, thermal conductivity and non-Newtonian rheological features, combining them with proper heat transfer modeling to accurately capture sagging dynamics.

The model was validated through a comprehensive experimental study using a desktop-scale PAM 3D printer to systematically evaluate the influence of four process parameters, i.e., nozzle temperature $T_{N}$ (190–200 °C), printhead speed $V_{P}$ (45–90 mm/s), screw peripheral speed N (30–60 rpm) and fan cooling activation (Off–On) on the maximum unsupported strand deflection.

This investigation yielded the following key findings:

- Active fan cooling is the paramount factor in reducing sagging during support-free 3D printing. It demonstrated a capacity to reduce bridge deflection by over 64.9%, underscoring how thermal management via forced convection far outweighs the influence of initial melt viscosity.
- Mass flow rate increases with screw speed, thus leading to higher bridge deflections, independently from extrusion temperature. Conversely, increasing the printhead speed reduces deflection by creating thinner, unsupported strands characterized by a lower volume-to-area ratio.
- A useful design criterion to realize support-free printing was identified: minimizing gravity-induced deflections requires the printing time to be less than the material’s cooling time to its melting point; this aspect is accurately predicted by the FE model.

Furthermore, the FE model captured the compensatory effects between parameters; for instance, at low extrusion temperature and high printhead speeds, the increased heat dissipation from fan activation was shown to nearly perfectly counteract the greater gravitational load from a higher mass flow rate.

The proposed validated FE model establishes a robust foundation to tune process parameters for reliable support-free printing in PAM.

Further work will focus on enhancing the model’s accuracy by addressing its key limitations. (i) The incorporation of material shrinkage during cooling and (ii) a more realistic model that accounts for the continuous increase in the viscous beam’s length corresponding to the printhead’s incremental movement are currently under study.

## Figures and Tables

**Table 1:** Experiment design for the investigation of support-free 3D printing.

$T_{N}({}^{\circ}C)$	$V_{p}(mm/s)$	N(rpm)	fan(−)
190	45	30	Off
190	45	30	On
190	45	60	Off
190	45	60	On
190	90	30	Off
190	90	30	On
190	90	60	Off
190	90	60	On
200	45	30	Off
200	45	30	On
200	45	60	Off
200	45	60	On
200	90	30	Off
200	90	30	On
200	90	60	Off
200	90	60	On

**Table 2:** Data-fitted Cross-WLF parameters of the PLA 3251D.

Cross-WLF Parameter	$A_{1}(-)$	$A_{2}(K)$	$D_{1}(Pa*s)$	$D_{3}(K/Pa)$	$T^{*}(K)$	n(−)	$\tau^{*}(Pa)$
Value	16.71	51.60	2.045 × 10^7^	0	373.15	0.3846	1.29 × 10^5^

**Table 3:** Mean experimental bridge deflection (${\bar{\delta }}_{exp}$) and mass flow rate ($\bar{\dot{m}}$) at varying process parameters.

$T_{N}({}^{\circ}C)$	$V_{p}(mm/s)$	N(rpm)	fan(−)	${\bar{\delta }}_{exp}(mm)$	$\bar{\dot{m}}(g/h)$
190	45	30	Off	3.67	97.46
190	45	30	On	1.95	36.46
190	45	60	Off	5.59	198.22
190	45	60	On	3.98	207.13
190	90	30	Off	3.17	122.40
190	90	30	On	1.93	69.80
190	90	60	Off	4.81	163.72
190	90	60	On	3.60	214.01
200	45	30	Off	5.28	133.18
200	45	30	On	3.54	108.58
200	45	60	Off	5.69	270.00
200	45	60	On	4.44	231.42
200	90	30	Off	3.65	115.12
200	90	30	On	3.04	112.90
200	90	60	Off	3.65	193.91
200	90	60	On	3.87	181.11

**Table 4:** Mesh-independence study for case $T_{N}$ = 190 °C, $V_{p}=\;$45 mm/s, N= 30 rpm and fan in Off state.

Number of Swept Elements	Number of Finite Elements (–)	Computational Time (min)	$\delta_{CFD}(mm)$	$\varepsilon_{rel}(\%)$
15	1350	2	3.4791	1.40
20	1800	5	3.5099	0.53
25	2250	7	3.5205	0.23
30	2500	8	3.5243	0.12
35	2700	9	3.5371	0.04
40	3150	10	3.5279	0.02
45	4050	12	3.5286	–

**Table 5:** Comparison of experimentally and numerically determined maximum deflections according to the process parameters of the experiment design.

$T_{N}$ (°C)	$V_{p}$ (mm/s)	N (rpm)	fan (−)	${\bar{\delta }}_{exp}$ (mm)	$\delta_{CFD}$ (mm)	$T_{N}$ (°C)	$V_{p}$ (mm/s)	N (rpm)	fan (−)	${\bar{\delta }}_{exp}$ (mm)	$\delta_{CFD}$ (mm)
190	45	30	Off	3.67	3.52 (4.09%)	200	45	30	Off	5.28	4.67 (11.55%)
190	45	30	On	1.95	2.09 (7.18%)	200	45	30	On	3.54	3.70 (4.51%)
190	45	60	Off	5.59	4.65 (16.81%)	200	45	60	Off	5.69	6.27 (10.19%)
190	45	60	On	3.98	4.00 (0.50%)	200	45	60	On	4.44	4.85 (9.23%)
190	90	30	Off	3.17	3.01 (5.04%)	200	90	30	Off	3.65	3.39 (7.12%)
190	90	30	On	1.93	2.04 (5.70%)	200	90	30	On	3.04	3.22 (5.92%)
190	90	60	Off	4.81	3.67 (23.70%)	200	90	60	Off	3.65	4.12 (12.88%)
190	90	60	On	3.60	3.00 (16.66%)	200	90	60	On	3.87	3.34 (13.70%)

**Table 6:** Time needed to reach 95% of final gravity-induced bridge deflection.

$T_{N}$ (°C)	$V_{p}$ (mm/s)	N (rpm)	fan (−)	$t_{95\%}$ (s)	$T_{N}$ (°C)	$V_{p}$ (mm/s)	N (rpm)	fan (−)	$t_{95\%}$ (s)
190	45	30	Off	1.41	200	45	30	Off	1.86
190	45	30	On	0.41	200	45	30	On	1.10
190	45	60	Off	2.70	200	45	60	Off	3.55
190	45	60	On	2.06	200	45	60	On	2.12
190	90	30	Off	0.95	200	90	30	Off	0.87
190	90	30	On	0.32	200	90	30	On	0.51
190	90	60	Off	1.20	200	90	60	Off	1.36
190	90	60	On	0.94	200	90	60	On	0.74

## Data Availability

The original contributions presented in this study are included in the article. Further inquiries can be directed to the corresponding author.

## Abbreviations

The following abbreviations are used in this manuscript:

AM	Additive Manufacturing
CFD	Computational Fluid Dynamics
FE	Finite Element
FFF	Fused Filament Fabrication
HTC	Heat Transfer Coefficient
MEX	Material Extrusion
PAM	Pellet Additive Manufacturing
PLA	Polylactic Acid
